# Mutation and evolutionary analyses identify *NR2E1-*candidate-regulatory mutations in humans with severe cortical malformations

**DOI:** 10.1111/j.1601-183X.2006.00277.x

**Published:** 2007-08

**Authors:** R A Kumar, S Leach, R Bonaguro, J Chen, D W Yokom, B S Abrahams, L Seaver, C E Schwartz, W Dobyns, A Brooks-Wilson, E M Simpson

**Affiliations:** † Centre for Molecular Medicine and Therapeutics and Child & Family Research Institute Vancouver, Canada; ‡ Department of Medical Genetics, University of British Columbia Vancouver, Canada; § Canada’s Michael Smith Genome Sciences Centre, British Columbia Cancer Agency Vancouver, Canada; ¶ Center for Molecular Studies, J.C. Self Research Institute, Greenwood Genetic Center Greenwood, SC, USA; †† University of Chicago Chicago, IL, USA

**Keywords:** Cortex, ‘fierce’ mice, mental retardation, microcephaly, nuclear receptor, *Tlx*

## Abstract

Nuclear receptor 2E1 (*NR2E1*) is expressed in human fetal and adult brains; however, its role in human brain–behavior development is unknown. Previously, we have corrected the cortical hypoplasia and behavioral abnormalities in *Nr2e1^−/−^* mice using a genomic clone spanning human *NR2E1*, which bolsters the hypothesis that *NR2E1* may similarly play a role in human cortical and behavioral development. To test the hypothesis that humans with abnormal brain–behavior development may have null or hypomorphic *NR2E1* mutations, we undertook the first candidate mutation screen of *NR2E1* by sequencing its entire coding region, untranslated, splice site, proximal promoter and evolutionarily conserved non-coding regions in 56 unrelated patients with cortical disorders, namely microcephaly. We then genotyped the candidate mutations in 325 unrelated control subjects and 15 relatives. We did not detect any coding region changes in *NR2E1*; however, we identified seven novel candidate regulatory mutations that were absent from control subjects. We used *in silico* tools to predict the effects of these candidate mutations on neural transcription factor binding sites (TFBS). Four candidate mutations were predicted to alter TFBS. To facilitate the present and future studies of *NR2E1*, we also elucidated its molecular evolution, genetic diversity, haplotype structure and linkage disequilibrium by sequencing an additional 94 unaffected humans representing Africa, the Americas, Asia, Europe, the Middle East and Oceania, as well as great apes and monkeys. We detected strong purifying selection, low genetic diversity, 21 novel polymorphisms and five common haplotypes at *NR2E1*. We conclude that protein-coding changes in *NR2E1* do not contribute to cortical and behavioral abnormalities in the patients examined here, but that regulatory mutations may play a role.

Genes with expression patterns and developmental functions consistent with a role in regulating neurogenesis and cortical size are suitable for studying the genetic basis of human brain development and evolution (Gilbert *et al.* 2005; Kornack & Rakic 1998; Rakic 1995). To date, only a limited number of genes have been identified that are expressed at sites of cortical neurogenesis that are known to regulate neural stem cells, forebrain size and behavior. One such gene is the nuclear receptor 2E1 (*Nr2e1*; previously *Mtll*, *Tailless*, *Tll* and *Tlx*), for which a clear role in mouse brain–behavior development makes it an excellent candidate for genetic studies of human abnormal brain–behavior development and evolution.

*NR2E1* is expressed in human fetal brain (Strausberg *et al.* 2002) and in mouse embryonic forebrain (Monaghan *et al.* 1995) and is also detected in the adult forebrains of humans and mice (Jackson *et al.* 1998; Shi *et al.* 2004). *Nr2e1* is required for normal temporal regulation of cortical neurogenesis during embryonic development and regulates proliferation and differentiation of neural progenitor cells in the embryonic and adult mouse cortex (Roy *et al.* 2002, Roy, 2004; Shi *et al.* 2004). Mice deleted for both copies of *Nr2e1* (*Nr2e1^−/−^*) show cortical hypoplasia, limbic system abnormalities, cognitive impairment, short stature, vision problems and abnormal social behaviors that include pathological violence (Christie *et al.* 2006; Kumar et al. 2004b; Land & Monaghan 2003; Miyawaki *et al.* 2004; Roy *et al.* 2002; Young *et al.* 2002).

Multiple additional lines of evidence support a role for *NR2E1* in human brain development. First, we have recently corrected the cortical and behavioral abnormalities of *Nr2e1^−/−^* mice using a genomic clone spanning the human *NR2E1* locus (Abrahams *et al.* 2005), providing robust evidence that human and mouse *NR2E1* are functionally equivalent in mice. Second, members of the nuclear receptor superfamily have been implicated in disorders of human brain and behavior, including *NR4A2* (Buervenich *et al.* 2000; Chen *et al.* 2001; Hering *et al.* 2004; Iwayama-Shigeno *et al.* 2003; Le *et al.* 2003; Smith *et al.* 2005) and the estrogen receptor (Westberg *et al.* 2003). Importantly, mutations in human and mouse *NR2E3,* a gene closely related to *NR2E1*, produce similar eye developmental abnormalities (Akhmedov *et al.* 2000; Haider *et al.* 2000), suggesting that human and mouse *NR2E1* mutations might also cause the same phenotype. Third, some individuals with cortical abnormalities have *de novo* interstitial deletions encompassing the *NR2E1* locus at 6q21. Chery *et al.* (1989) report a *de novo* interstitial deletion of 6q21 in a male with moderate microcephaly, facial dysmorphism and psychomotor retardation (Chery *et al.* 1989). In addition, patient 2 reported by Hopkin *et al.* (1997) has an interstitial deletion that includes 6q21 and presents with severe intrauterine growth retardation and severe congenital microcephaly (Hopkin *et al.* 1997).

*NR2E1* hypomorphic mutations could underlie human cortical malformations. Mice deleted for a single copy of *Nr2e1* (*Nr2e1^+/−^*) show premature neurogenesis during early corticogenesis that results in reduced neuron numbers that are intermediate to that produced in *Nr2e1^+/+^* and *Nr2e1^−/−^* mice (Roy *et al.* 2004), providing strong support for dosage sensitivity for *Nr2e1* during cortical development. Support for a hypomorphic mechanism is also provided by studies in mice that are double heterozygotes for *Nr2e1* and *Pax6*, which result in altered regionalization of the cerebral cortex (Stenman *et al.* 2003). Mice heterozygous for either *Nr2e1* or *Pax6* alone do not show alterations in cortical gene expression at the pallial–subpallial boundary, indicating that normal cortical regionalization at this boundary involve a genetic interaction between *Pax6* and *Nr2e1* (Stenman *et al.* 2003). Importantly, human cortical malformations are known to result from *PAX6* haploinsufficiency (Sisodiya *et al.* 2001). In addition, Glaser et al. (1994) describe a newborn boy with homozygous mutations of *PAX6* that results in severe congenital microcephaly and polymicrogyria (Glaser *et al.* 1994). Taken together, mouse and human genetic studies support the proposal that some human cortical disorders may involve a single- or a multigene mechanism involving *NR2E1* null or hypomorphic mutations.

In this study, we report the first genetic analyses of *NR2E1* in patients. To test the hypothesis that humans with abnormal cortical development and mental retardation may have null or hypomorphic mutations in *NR2E1*, we searched for candidate mutations by sequencing the complete coding region, 5′- and 3′-untranslated (UTR), splice site, proximal promoter and evolutionarily conserved non-coding regions in 56 unrelated patients with unexplained congenital microcephaly, a neurodevelopmental disorder characterized by marked reduction in cortical size that may result from failure of neurogenesis (Dobyns 2002; Mochida & Walsh 2001). We genotyped candidate mutations in ethnically matched control subjects that included 137 Africans and 188 Europeans. To guide the present and future studies of *NR2E1*, we also elucidated its molecular evolution, genetic diversity, haplotype structure and linkage disequilibrium by sequencing an additional 94 unaffected humans representing Africa, the Americas, Asia, Europe, the Middle East and Oceania, as well as chimpanzee, gorilla, orangutan and macaque.

## Materials and methods

### Human and non-human primate samples

Approval for this study was obtained from The University of British Columbia (Certificate of Approval # C99-0524), Child & Family Research Institute of British Columbia (Certificate of Approval # W00-0005) and the Department of Medical Genetics (Certificate of Approval #6-3-20). The research followed the Canada’s Tri-Council Statement on ‘Ethical Conduct for Research Involving Humans’ (sections 2.5–2.7). We studied 56 unrelated patients with congenital microcephaly (with or without simplified gyral patterns) and additional features resembling *Nr2e1^−/−^* mice, including short stature, vision problems, cognitive impairment and abnormal social behaviors. Patient demographic and clinical data are reported in Table 1. For a subset of patients, unaffected and affected family members that included 14 parents and four siblings were also studied. The following control subjects without severe cortical malformations or known behavioral problems were studied: (1) 110 individuals of African descent obtained from the Coriell Cell Repository (http://coriell.umdnj.edu/); (2) 27 individuals of African descent obtained from Dr M. R. Hayden (University of British Columbia, Vancouver, Canada); (3) 94 Caucasians obtained from the Coriell Cell Repository (http://coriell.umdnj.edu/); and (4) 94 Caucasian patients diagnosed with Gilbert syndrome. For genetic diversity and molecular evolutionary studies, we examined an additional 94 ethnically diverse unaffected humans, who included African (African-American, Mbuti, Biaka), American (Cheyenne, Mayan, Quechua, Karitiana), Asian (Indo-Pakistani, Chinese, Japanese), European (Russian, Italian, Northern European, Icelandic), Middle Eastern (Ashkenazi Jewish, Druze Arab) and Oceanic people (Pacific and Melanesian). Ethnically diverse DNA samples were obtained from the Coriell Cell Repository (http://coriell.umdnj.edu/) and do not overlap with any of the Coriell ethnically matched control subjects described above. Great ape tissues were obtained from Dr E. Eichler (University of Washington, Seattle, USA). DNAs (three chimpanzees, three gorillas, three orangutans) were isolated from either lymphoblasts or fibroblasts using the Gentra Puregene kit (Minneapolis, MN, USA). Macaque DNAs (two rhesus macaques, two Japanese macaques) were obtained from Oregon Regional Primate Research Center (Beaverton, OR, USA).

**Table 1:** Demographic and clinical information on patients with cortical malformations

Patient ID	Ethnicity	Sex	Brain abnormality	MR	Seizures	Psychosis	Stature	Vision problems	Other
CMS 3226	b	m	mic	Yes	Yes	No	Short	u	−
CMS 5041	b	m	mic	Yes	No	Yes	Normal	u	−
CMS 5811	b	m	mic	Yes	Yes	Yes	Short	u	−
CMS 5162	w	m	mic	Yes	Yes	Yes	Short	u	−
CMS 4775	w	m	mic	Yes	Yes	No	Short	u	−
CMS 5207	b	m	mic	Yes	Yes	No	Normal	Yes	−
CMS 5315	b	m	mic	Yes	Yes	No	Short	u	−
CMS 7456	u	m	mic	Yes	No	Yes	u	u	−
CMS 5538	b	m	mic	Yes	No	Yes	Normal	u	−
CMS 5838	b	m	mic	Yes	No	Yes	Normal	u	−
CMS 5151	w	m	mic	Yes	No	Yes	Normal	u	−
12856	u	m	mic	Yes	No	Yes	Normal	u	−
17763	w	m	mic	Yes	Yes	Yes	Normal	u	−
8348	b	m	mic	Yes	Yes	Yes	u	u	−
11362	w	m	mic	Yes	No	Yes	Normal	u	−
29494	w	m	mic	Yes	Yes	No	Short	u	−
LP95-042a2	w	m	mic msg	Severe	Yes	u	u	No	Early death
LP97-105	u	f	mic msg xax	u	u	u	u	u	−
LP98-038a1	w	f	mic msg	Moderate	No	No	u	No	−
LP98-052	w	m	mic msg pmg	Severe	Yes	u	u	No	Early death
LP98-095	w	f	mic msg	Mild	No	No	u	No	−
LP99-035	w	m	mic msg	Severe	u	u	u	u	Jejunal
LP99-0100a1	w-me	f	mic msg	Severe	Yes	u	u	u	−
LP99-156	w	m	mic msg bch	Severe	u	u	u	u	Early death
LR00-025	u	m	mic msg	u	Yes	u	u	u	−
LR00-144	w	m	mic msg	Severe	Yes	u	u	u	Early death
LR00-182a1	w-ash j	f	mic msg	Severe	Yes	u	Normal	No	−
LR00-188	w-me	m	mic msg	u	u	u	u	u	−
LR00-196	u	m	mic msg acc	Severe	u	u	u	u	Jejunal
LR00-204	u	f	mic msg	Severe	u	u	u	u	Jejunal
LR01-068	w	f	mic msg	u	u	u	u	u	−
LR01-099	u	f	mic msg bch xax acc	Severe	Yes	u	u	Optic atrophy	−
LR01-148	u	f	mic msg bch xax	Severe	Yes	u	u	No	−
LR01-171	w-me	m	mic msg	Mild	No	No	Normal	No	−
LR01-194	w	m	mic msg bch acc	Severe	Yes	u	u	u	−
LR01-224	w	m	mic msg xax	Moderate	No	No	Normal	Yes	−
LR01-265	w	f	mic msg	Severe	Yes	u	u	No	−
LR01-271	w	f	mic msg acc	u	Yes	u	u	u	−
LR01-314	u	m	mic msg	u	Yes	u	Normal	No	−
LR01-338	w	f	mic msg	u	No	u	u	No	−
LR01-356	w-me	m	mic msg bch	u	u	u	u	u	−
LR02-005	w	f	mic msg xax	u	u	u	u	u	−
LR02-016a3	w	u	mic msg bch	u	u	u	u	u	−
LR02-046	w	f	mic msg acc	u	No	u	u	u	−
LR02-080	u	m	mic msg	u	u	u	u	u	−
LR02-085	w	f	mic msg	Mod-severe	Yes	u	u	Amblyopia	−
LR02-112	u	f	mic msg xax	u	No	u	u	u	−
LR02-153	w-me	f	mic msg bch	u	u	u	u	u	−
LR02-154a1	w	f	mic msg xax	u	Yes	No	u	Sclerocornea	−
LR02-171	u	m	mic msg acc	u	u	u	u	micr scl	−
LR02-304	u	m	mic msg	u	No	u	u	u	−
LR02-421	w	m	mic msg	dd	u	u	u	u	−
LR03-059	u	f	mic msg xax	dd	Yes	No	u	u	−
LR03-184a1	u	m	mic msg bch	Severe	Yes	No	u	u	−
LR03-277	u	m	mic msg xax	Severe	u	u	u	u	−
gEMS594	u	m	mic	Severe	u	u	Short	Micropthalmia	−

*Ethnicity:* b, black; w, white; w-me, white-Middle Eastern; w-ash j, white Ashkenazi jewish; u, unknown.

*Sex:* f, female; m, male.

*Brain abnormality:* acc, agenesis of the corpus callosum; bch, brainstem-cerebellar hypoplasia; mic, microcephaly; msg, microcephaly with simplified gyral pattern; pmg, polymicrogyria, xax, enlarged extra-axial space.

*MR:* MR, mental retardation; note that for some patients, MR was scored as being present (i.e. ’yes‘) whereas for other patients the severity of MR was noted (i.e. mild, moderate, moderate-severe (Mod-severe), or severe); dd, developmental delay.

*Vision problems:* micr scl, micropthalmia and sclerocornea.

*Other:* jejunal, jejunal atresia;−, no other phenotypes noted.

u, unknown.

### DNA amplification and sequencing

We sequenced *NR2E1* using DNA amplicons generated from 20 polymerase chain reaction (PCR) assays that covered the coding region (1146 bp), complete 5′- and 3′-UTRs (1973 bp) and exon-flanking regions including consensus splice sites (1719 bp). In addition, we sequenced six evolutionarily conserved non-coding regions including proximal promoter (1528 bp) as previously described (Abrahams *et al.* 2002). Human genomic *NR2E1* sequence AL078596 (http://www.ncbi.nlm.nih.gov/) was used as the reference sequence. Polymerase chain reactions were performed in a 96-well microtitre plate thermal cycler. Polymerase chain reactions were prepared in a total volume of 20 μl using 10 ng of genomic template and the following reagents from Invitrogen (Burlington, Ontario, Canada): 1× buffer, 1 mm MgSO_4_, 0.2 mm dNTPs, 0.5 mm primer [each of forward and reverse (Table 2)] and 0.0125 units *Pfx* polymerase. Thermal cycling was performed as follows: 30 cycles, 94°C for 2 min, annealing T (58–63°C) for 30 seconds, 68°C for 1 min. Polymerase chain reaction products were purified using magnetic beads from Agencourt Bioscience Corporation (Beverly, MA, USA) as per manufacturer’s instructions. Non-human primate sequencing reactions used 10–20 ng of DNA under similar conditions. Sequencing reactions performed in 384-well plates were as follows: BD Ready Rxn Mix V3 (0.54 μl), 5× Reaction Buffer (0.43 μl), 5 μm Primer (0.26 μl; Table 2), 0.2 μm 18 MΩ ddH20 (0.77 μl) and DNA (5–100 ng). Sequences were visually inspected and scored by at least two individuals using either Consed (Gordon *et al.* 1998) or Sequencher (Gene Codes, Ann Arbor, MI, USA). Every human variant that was identified only once (i.e. singletons) was confirmed by repeating the PCR and sequencing process. The CA-repeat assay (D6S1594; GenBank Accession Z52880) was prepared in a total volume of 15 μl using 10–50 ng of genomic template and the following reagents from Invitrogen: 1× buffer, 2.5 mm MgSO_4_, 0.25 mm dNTPs and 0.04 units *Pfx* polymerase. Primers (0.5 mm) were fluorescently labeled with FAM (ABI, Foster City, CA, USA). Post-PCR products were diluted 1:30 with ddH_2_O and 1 μl was combined with a 9.5 μl mix of formamide and Gene Scan™ 400HD ROX as per manufacturer’s instructions (ABI). Samples were denatured at 95°C for 5 min and placed on ice until loaded onto the ABI 3100 Genetic Analyzer (ABI). Polymerase chain reaction fragments were analyzed using Gene Mapper 3.0 (ABI).

**Table 2:** Polymerase chain reaction primers used to amplify *NR2E1* sequences

	Forward*		Reverse†	
		
Assay	Name	Sequence	Name	Sequence
CE11A	oEMS1988	5′-TACGCCTTAAATCCGAGGTC-3′	oEMS1989	5′-CGATCAAGCATGGTGTCAAG-3′
CE12A	oEMS1990	5′-TGACACCGAGTCTGGAGAAA-3′	oEMS2031	5′-GTCGCCTCCATTATCTGCAC-3′
CE13A	oEMS1994	5′-CAGCTCTGCTTGGGGGAAG-3′	oEMS1995	5′-AAAACGCTTTTCCCCCTCT-3′
CE14A	oEMS1998	5′-TCCTTCTTGCCGTGAAATATAC-3′	oEMS2032	5′-GGAAAACTAGATTGCTGGGAAAT-3′
5′-UTRa	oEMS2033	5′-CCAGGGACGCCCTATTCC-3′	oEMS2034	5′-GAGGAAGAAGGAAGAACAGCA-3′
5′-UTRb	oEMS2035	5′-CCCACACTCTGCATGCCTAT-3′	oEMS2036	5′-GACAGGTGGGTGTCAGTCG-3′
Exon1	oEMS2037	5′-TGTGTCCATATCAAGCAGCA-3′	oEMS2038	5′-CTCCACGAAATGCTCCAACT-3′
CE17B	oEMS2011	5′-GGAGAGCAGAGCGATGTCAC-3′	oEMS2012	5′-TCACGAGACAAGCTGGTTGA-3′
CE19B	oEMS2013	5′-CCTCCCACAGCACAATCTC-3′	oEMS2016	5′-GTCCCAGACTCGTCTCAGGT-3′
Exon2	oEMS1966	5′-TTCGGTGCTAATCCCTTCAG-3′	oEMS1967	5′-AGAGGAAGGGAGAGGTCAGG-3′
Exon3	oEMS1968	5′-GGACTGGCCCTCTTGAAGTA-3′	oEMS1969	5′-TCCCAGCATCTGGAAAGAAG-3′
Exon4	oEMS1970	5′-CTCCCTCAGATTCCCTCTCC-3′	oEMS2039	5′-AACTGGGTGCGTCCCTCT-3′
Exon5	oEMS1972	5′-TACCCACCAATGTCAACTGC-3′	oEMS1973	5′-AACCCACAGGAAGAAGCAAG-3′
Exon6	oEMS1974	5′-TGGGAAAATAAGGGAAAGCTAGA-3′	oEMS1975	5′-ATTTAAATAACAATGCAAGCAGTCA-3′
Exon7	oEMS1976	5′-CTTTCATACAATATAGCCGGTTTACA-3′	oEMS1977	5′-AACATGCAGGTTCCCATAGC-3′
Exon8	oEMS1978	5′-GATTACAGACACATGCCACCAT-3′	oEMS1979	5′-CACCCACCCTGAGAGATAGG-3′
Exon9	oEMS2040	5′-TTCAAGTGTAAGACGTTAGTTTCCA-3′	oEMS2041	5′-CTGTGGCAACCCCCAGTT-3′
3′-UTRa	oEMS2042	5′-AAAGCATTCCAGTAGCTATGACC-3′	oEMS2043	5′-GTTGCCTGGCCTATGGTATT-3′
3′-UTRb	oEMS2044	5′-CATTATTAAGTGGCCTTCAGAACT-3′	oEMS2045	5′-CAGTTTTCGGAAAGGCATTG-3′
3′-UTRc	oEMS2046	5′-CCAGACAGGAAACGAATATGG-3′	oEMS2047	5′-CCTTGTTTCTGGTGGGTGAG-3′

* 5′-TGTAAAACGACGGCCAGT-3′ sequence (-21M13F) was added to the 5′ end of each forward primer to facilitate sequencing.

† 5′-CAGGAAACAGCTATGAC-3′ sequence (M13R) was added to the 5′ end of each reverse primer to facilitate sequencing.

### Transcription factor binding site (TFBS) analyses

To predict whether genetic variants at *NR2E1* (i.e. candidate mutations, polymorphisms and human-specific nucleotides) alter experimentally validated consensus-binding sequences for neural transcription factors, we performed TFBS analyses using MatInspector (Quandt *et al.* 1995). We analyzed the minor and major alleles at each variant site together with 50 bp of surrounding sequence using the Optimized Matrix Similarity thresholds. We focused specifically on transcription factors with brain-relevant roles that include cortical patterning, neural cell proliferation and differentiation, neuronal apoptosis, neuronal survival and synaptic plasticity.

### Evolutionary, nucleotide diversity and genetic differentiation analyses

The following standard measures of genetic diversity were calculated using DnaSP version 3.0 (Rozas & Rozas 1999): *S* (the number of segregating sites); and θ_W_ and π (nucleotide diversity). The following statistical tests of selection were performed using DnaSP version 3.0 (Rozas & Rozas 1999): Tajima’s *D*-test (which compares the number of nucleotide polymorphisms (θ_W_) with the mean pairwise difference between sequences (π); Fu and Li’s D^*^ (which compares the number of derived nucleotide variants observed only once in a sample with the total number of derived nucleotide variants); Fu and Li’s F^*^ (which compares the number of derived nucleotide variants observed only once in a sample with the mean pairwise differences between sequences) and Fay and Wu’s *H* (which compares the number of derived nucleotide variants observed only once in a sample with the mean pairwise differences between sequences). Non-human primate outgroups were used to infer the ancestral and derived states of human variants. The *P* values for Tajima’s *D* and Fay and Wu’s *H* were estimated from 10 000 coalescent simulations of an infinite site locus that conditioned on the sample size. Human and non-human primate sequence data were aligned using MEGA version 3.0 (Kumar *et al.* 2004c) and human-specific variants were identified visually and confirmed by at least two individuals.

### Haplotype and linkage disequilibrium reconstruction

We reconstructed haplotypes and estimated their frequencies by implementing PHASE (V. 2.0). We calculated haplotype diversity for each population as 2*n*(1−Σ*x_i_*^2^)/(2*n*−1), where *x_i_* is the frequency of haplotype *i* and *n* is the sample number. Pairwise linkage disequilibrium (LD) between each common SNP was computed as |*D’*| and *r^2^* using DnaSP version 3.0 (Rozas & Rozas 1999). We did not analyze the indels because gaps are excluded from the LD analyses (Rozas & Rozas 1999). Significance of LD was tested using Fisher’s exact test after Bonferroni adjustment for multiple tests.

## Results

### Candidate NR2E1 mutations identified in patients with cortical abnormalities

In total, we generated approximately 368 220 bp of *NR2E1* sequence data. We did not detect any synonymous or non-synonymous coding variants. Nine out of the 56 patients (16%) were homozygous across all sites sequenced, which spanned 25.5 kb. We identified 11 patients harboring 15 novel non-coding variants (i.e. variants that have not been previously reported (http://www.ncbi.nlm.nih.gov/projects/SNP/; Build 124) (Table 3). Each of these variants (herein referred to as ‘patient variants’) was present in the heterozygote state. Thirty-three percent of the patient variants resided within the proximal promoter, 33% within a UTR and 33% within intronic sequence. Transitions and transversions accounted for 47% and 53% of all variants, respectively.

**Table 3:** Characterization of 15 *NR2E1* patient variants in families and control subjects

			Genotype§				
							Frequency of patient variant in control chromosomes¶
Patient ID*	Location†	Nucleotide variant‡	Patient	Unaffected father	Unaffected mother	Sibling	
LR00-144	CE11A	g.-2945A>G	A/G	A/A	A/G	n/a	0/330 (0%)
LR00-144	PPR	g.-1767G>T	G/T	G/G	G/T	n/a	0/518 (0%)
LR00-144	3′-UTR	g.21502TG>C	T/C	T/C	T/T	n/a	0/344 (%)
LR03-184a1	PPR	g.-1431C>A	C/A	C/C	C/A	C/C	6/528 (1.1%)
LR03-184a1	Intron 1	g.151T>A	T/A	T/T	T/A	T/T	6/350 (1.7%)
LR00-204	PPR	g.-1453C>G	C/G	C/C	C/G	n/a	1/528 (0.2%)
LR00-204	Intron 5	g.11559C>T	C/T	C/T	C/C	n/a	2/550 (0.4%)
LR03-277	3′-UTR	g.21762C>A	C/A	C/A	C/C	n/a	1/352 (0.3%)
LR03-277	3′-UTR	g.21796G>A	G/A	G/G	G/A	n/a	1/352 (0.3%)
LR02-304	CE12A	g.-1726C>A	C/A	C/A	C/C	n/a	0/528 (0%)
LP98-052	PPR	g.-1453C>G	C/G	C/G	C/C	n/a	1/528 (0.2%)
CMS5151	5′-UTR	g.-555C>T	C/T	n/a	n/a	n/a	2/540 (0.4%)
8348	Intron 3	g.8213T>C	T/C	n/a	n/a	n/a	0/146 (0%)
12856 XS	Intron 7	g.14617A>C	A/C	n/a	n/a	n/a	1/558 (0.2%)
LR01-194	Intron 7	g.14718C>T	C/T	C/C	C/T	n/a	1/558(0%)
LR01-148	3′-UTR	g.20765C>A	C/A	n/a	n/a	n/a	0/362 (0%)

* Note that patients LP98-052 and LR00-204 both harboured identical variants (i.e. g.-1453C>G). Thus, a total of 15 novel variants were identified.

† PPR, proximal promoter region (defined as a 2.0-kb region upstream of the initiator Met codon); CE, evolutionary conserved element within PPR (as described in Abrahams *et al.* 2002); UTR, untranslated region.

‡ g, genomic; numbering based on Antonarakis and the Nomenclature Working Group [1998], where A of the initiator Met codon in exon 1 is denoted nucleotide +1. Human genomic *NR2E1* sequence: NCBI AL078596.

§ Sibling of LR03-184a1 is affected with microcephaly with simplified gyral pattern.

¶ numbers represent the total number of successfully sequenced chromosomes and not the total number of chromosomes screened.

n/a, not available.

Four patients harbored multiple patient variants, including patients LR00-44, LR03-184a1, LR00-204 and LR03-277, in whom we identified three, two, two and two patient variants, respectively. Patients LR00-204 and LP98-052 both harbored the g.-1453C>G substitution.

We amplified and sequenced the regions corresponding to the 15 novel patient variants in 15 additional family members (Table 3). Fourteen parents were available for typing for 12 of the 15 novel patient variants, including both parents for the two unrelated patients having the identical g.-1453C>G patient variant. In all 12 cases, at least one unaffected parent harbored the patient variant, indicating that none of these variants were *de novo*. For four patient variants, parents were unavailable for typing; therefore, we cannot exclude the possibility that these variants are *de novo*. An affected sibling was studied for both patient variants identified in patient LR03-184a1; neither of the two variants was identified in the sibling, suggesting that the two patient variants do not predict the cortical phenotypes in these siblings.

We amplified and sequenced the regions corresponding to the 15 novel patient variants in ethnically matched controls of African (274 chromosomes) and European (376 chromosomes) descent. If the ethnicity of the patient was unknown, the patient variants were genotyped in chromosomes of African and European descent (650 chromosomes). None of the control subjects were reported to have cortical malformations. Of the 15 novel variants identified in the patients, seven (g.-2945A>G, g.-1767G>T, g.-1726C>A, g.8213T>C, g.14718C>T, g.20765C>A and g.21502T>C) were not detected in any control subject (Table 3). These seven patient variants will now be referred to as ‘candidate mutations’. Three of the seven candidate mutations (g.-2945A>G, g.-1767G>T and g.21502T>C) were identified in patient LR00-144: two of these (g.-2945A>G and -1767G>T) were maternal and one (g.21502T>C) was paternal. Consequently, patient LR00-144 is a compound heterozygote for *NR2E1* mutations. Importantly, both g.-2945A>G and g.-1767G>T reside within the proximal promoter (PPR) and g.21502T>C resides within the 3′-UTR, which makes each of these variants reasonable candidates for putatively regulatory hypomorphic mutations. The four remaining candidate mutations were identified individually in unrelated patients. Two of these reside within putative regulatory regions (g.-1726C>A in a 100-bp element in the PPR that is conserved between mouse and human; and g. 20765C>A that resides in the 3′-UTR); the remaining two candidate mutations were identified in intronic regions outside the consensus splice site.

Two additional patients were compound heterozygotes for patient variants of *NR2E1*. Patient LR00-204 harbored g.-1453C>G (maternal) and g.11559C>T (paternal), each present in the general population at 0.2% and 0.4%, respectively. Patient LR03-277 harbored g.21762C>A (paternal) and g.21796G>A (maternal), both present in the general population at 0.3%. We did not identify a single control subject bearing either g.-1453C>G / g.11559C>T or g.21762C>A / g.21796G>A allelic pairs. We therefore consider these variants as candidates for rare functional polymorphisms.

### Predicted alterations of consensus transcription factor binding sites by NR2E1 candidate mutations

To predict the impact of the seven candidate mutations on transcription factor binding, we performed *in silico* analyses on experimentally-validated consensus sequences for TFBS. We restricted our analyses to transcription factors expressed in the brain. Of the seven candidate mutations, four (g.-1767G>T, g.-1726C>A, g.8213T>C, g.14718C>T) were predicted to create or abolish binding of transcription factors known to have roles in neuronal proliferation and survival, cortical patterning, neuronal differentiation and synaptic plasticity (Table 4). Of the four functional polymorphisms, one (g.-1453C>G) was predicted to create binding of two neural transcription factors (Table 4).

**Table 4:** *NR2E1* patient variants predicted to alter neural transcription factor consensus-binding sites

Variant type	Nucleotide variant	Location	Transcription factor binding site	Transcription factor (s)	Role in brain	Orthologous major allele in other species*				
						Human	Apes	Macaque	Mouse	Fugu
Candidate mutation	g.-1767G>T	PPR	Created	*lA-1*	Regulator of neuronal development	G	G	G	na	na
			Created	*NRSE*	Repressor of multiple neuronal genes					
Candidate mutation	g.-1726C>A	CE12A	Abolished	*SP1*	Regulator of neuronal survival	C	C	C	C	C
Candidate mutation	g.8213T>C	Intron 3	Abolished	*OCT-1*	Regulator of neuronal differentiation	T	T	T	T	T
			Created	*BRN-5*	Regulator of neuronal differentiation					
			Created	*PAX-6*	Regulator of neuronal Proliferation and fate					
Candidate mutation	g.14718C>T	Intron 7	Created	*TBX5*	Regulator of eye morphogenesis	C	N	na	na	na
Candidate mutation	g.2945A>G	CE11A	No effect	n/a	n/a	A	A	A	A	na
Candidate mutation	g.20765C>A	3′-UTR	No effect	n/a	n/a	C	C	C	C	na
Candidate mutation	g.21502T>C	3′-UTR	No effect	n/a	n/a	T	T	T	na	na
Functional polymorphism	g.-1453C>G	PPR	Created	*EGR3*	Regulator of synaptic plasticity	C	C	C	na	na
			Created	*NUDR*	Regulator of 5-HT1A receptor in neurons					
Functional polymorphism	g.11559C>T	Intron 5	No effect	n/a	n/a	C	C	C	C	na
Functional polymorphism	g.21762C>A	3′-UTR	No effect	n/a	n/a	C	C	C	na	na
Functional polymorphism	g.21796G>A	3′-UTR	No effect	n/a	n/a	G	−	A	na	na

See Table 3 for definitions.

*EGR3*, Early growth response gene 3 product.

n/a, not applicable.

* apes include chimpanzee, gorilla, and orangutan. na, ortholgous region does not align with human sequence (in the case of ‘Macaque’, this region was sequenced but does not align; N, refers to nucleotide variability among apes; −, sequence data not available.

To determine whether functional constraint may exist at the sites corresponding to the seven candidate mutations, we determined the orthologous major allele at each of the sites in chimpanzee, gorilla, orangutan, macaque, mouse and *Fugu*. Notably, in two instances (g.-1726C>A and g.8213T>C), the major human nucleotide was conserved to *Fugu* (Table 4). The absence of nucleotide variability at these two non-coding sites between human and *Fugu*, which are separated by 900 million years (Kumar & Hedges 1998), suggests strong functional constraint and supports the proposal that these sites may represent putative regulatory regions.

To determine whether the *NR2E1* candidate mutations may reside within *cis*-acting UTR motifs that are known to be critical for many aspects of gene expression and regulation (Mignone *et al.* 2002), we searched for the presence of experimentally validated functional motifs in the 5′- and 3′-UTR of *NR2E1* using UTRscan (Mignone *et al.* 2005). We identified three motifs in the 5′-UTR (15-LOX-DICE, IRES, Brd-Box) and two in the 3′-UTR (IRES, Brd-Box); however, none of these motifs included a candidate mutation. To determine whether any of the candidate mutations may alter 3′-UTR binding for microRNAs (miRNA), which are known to regulate genes (Bartel 2004), we aligned the 3′-UTR of *NR2E1* against known miRNA motifs (Xie *et al.* 2005). We detected two motifs; however, neither included a candidate mutation.

### Strong purifying selection and low nucleotide diversity at NR2E1 in ethnically diverse humans

Genetic diversity and molecular evolutionary studies of neural genes in humans and non-human primates represent powerful tools for understanding cortical development (Enard & Paabo 2004; Gilbert *et al.* 2005). We therefore sought to gain additional insight into the extent and patterns of genetic variation at *NR2E1* by systematically resequencing the same coding and non-coding regions as described above in 94 unaffected, ethnically diverse humans representing Africa, the Americas, Asia, Europe, the Middle East and Oceania; none of these humans was studied as part of the data set used as controls in our previous analyses. In addition, we studied *NR2E1* in chimpanzee, gorilla, orangutan, Japanese macaque and rhesus macaque. The human sample size chosen was sufficient to detect alleles with minor allele frequencies of 10% or greater with 90% power.

We did not detect a single non-synonymous or synonymous change in the coding region of any human sample. We also did not detect a single non-synonymous change in any non-human primate sample (2–3 individuals from five species). The complete lack of synonymous variation among humans and the complete absence of non-synonymous variation between humans and non-human primates suggests that *NR2E1* has experienced strong functional constraint (i.e. purifying selection).

**Figure 1:**
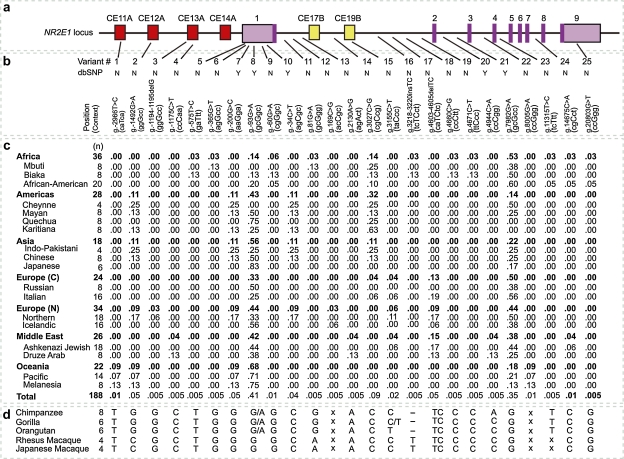
**A few common and many rare *NR2E1* variants detected in human populations representative for global diversity.** (a) Functional and putatively functional regions of *NR2E1* were resequenced, including coding (dark purple boxes), 5′- and 3′-untranslated (light purple boxes), and human non-coding regions that are conserved (Abrahams *et al.* 2002) in mouse (CE-A; red boxes) and mouse and *Fugu* (CE-B; yellow boxes). (b) A total of 26 variants was identified (variants 1–25 and CA-repeat, see Fig. 2). The nucleotide in the first position represents the human major (i.e. consensus) allele. Numbering based on Antonarakis and the Nomenclature Working Group (Antonarakis 1998), where A of the initiator Met codon in exon 1 is denoted nucleotide +1. Human genomic *NR2E1* sequence: AL078596 (http://www.ncbi.nlm.nih.gov/). Variants catalogued in dbSNP (‘Y’) are distinguished from those that are newly discovered here (‘N’). The DNA context of each variant is shown. (c) The number of chromosomes surveyed (*n*) and minor allele frequencies for each variable site are indicated for 18 world populations. (d) The corresponding chimpanzee, gorilla, orangutan, rhesus macaque, and Japanese macaque alleles are indicated. ‘x’ indicates that no sequence was obtained due to failed PCR or sequencing reaction. ‘−’ indicates that no corresponding nucleotide was present at that position in the non-human primate.

In this ethnically diverse sample, we observed a total of 25 non-coding variants (Fig. 1a; variants 1–25). Twenty-three of the 95 subjects (24%) were homozygous across all sequenced sites. Twenty of the 25 variants were novel (http://www.ncbi.nlm.nih.gov/projects/SNP/; dbSNP Build 124) (Fig. 1b). None of the seven candidate mutations identified in patients were detected in this unaffected ethnically diverse human panel. Consequently, if we include control subjects of mixed ethnic origin into our analyses of candidate mutations, the total number of unaffected chromosomes not harboring candidate mutations is as follows: g.-2945A>G (518 chromosomes), g.-1767G>T (706 chromosomes), g.-1726C>A (716 chromosomes), g.8213T>C (334 chromosomes), g.14718C>T (746 chromosomes), g.20765C>A (550 chromosomes) and g.21502T>C (532 chromosomes).

**Figure 2:**
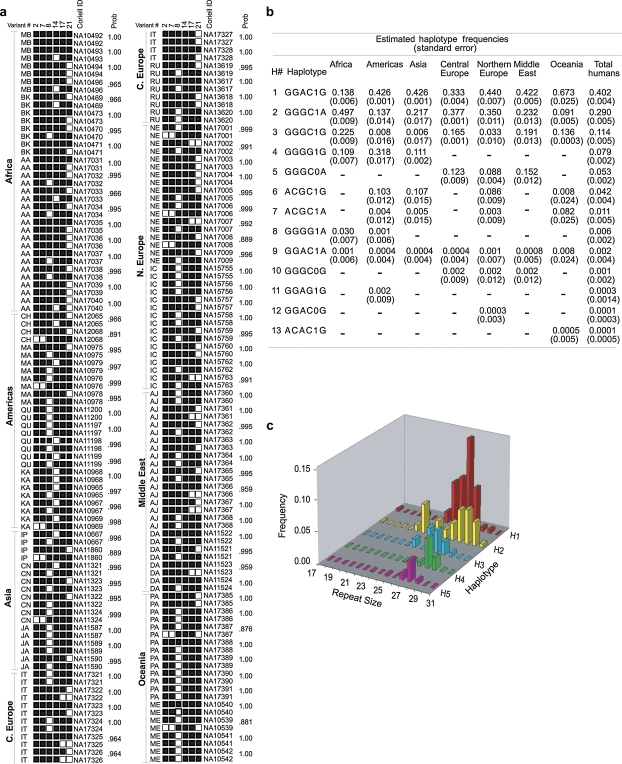
**Five common SNP-based *NR2E1* haplotypes account for the majority of chromosomes examined for global diversity.** (a) The SNP-based haplotypes for both chromosomes of every individual are illustrated. Each row represents one chromosome. Each column represents one variable site, the number of which is indicated above each column (see Fig. 1b). Black boxes indicate the major allele; white boxes represent the minor allele. Coriell Cell Repositories ID codes are indicated. ‘SNP’ refers to single nucleotide polymorphism (i.e. single nucleotide substitutions with minor allele frequencies ≥1%). ‘Prob’ is the probability of haplotype assignment, where 1.00 = 100% probable (i.e. individual is either homozygous at all sites or a heterozyote for only one site). MB, Mbuti; BK, Biaka; AA, African-American; CH, Cheyenne; MA, Mayan; QU, Quechua; KA, Karitiana; IP, Indo-Pakistani; CN, Chinese; JA, Japanese; IT, Italian; RU, Russian; NE, Northern European; IC, Icelandic; AJ, Ashkenazi Jewish; DA, Druze Arab; PA, Pacific Islanders; ME, Melanesian. (b) Estimated population haplotype frequencies of the 13 most frequent SNP-based *NR2E1* haplotypes. ‘−’ indicates that the haplotype is absent from the population. ‘1’ and ‘0’ represent present and absence of TC indel, respectively. (c) The frequency (*y*-axis) of the CA-repeat allele (*x*-axis) with the five most common *NR2E1* haplotypes (*z*-axis) is plotted for the global diversity population.

We determined the frequencies of all 25 variants in each ethnic group. Only six of the 25 variants (numbers 2, 7, 8, 14, 17 and 21) were common (i.e. minor allele frequency (MAF) ≥5%) (Fig. 1c). For each human variant, we also inferred the ancestral and derived states by comparing it to the orthologous non-human primate sequence (Fig. 1d). Interestingly, chimpanzee, gorilla and orangutan were all polymorphic for the same G > A transition (variant 8) observed in humans. This is the first report of a human polymorphic site that is also polymorphic for the same alleles across these three great apes.

We estimated the levels of human nucleotide diversity by computing θ_W_, which is based on the proportion of segregating sites (*S*) in a population and π, which is based on the average number of nucleotide differences per site between two sequences randomly drawn from the population (Hartl 1997) (Table 5). The total human estimates for θ_W_ (5.7 × 10^−4^± 0.17 × 10^−4^) and π (2.6 × 10^−4^± 0. 20 × 10^−4^) for *NR2E1* fall at the lower 20% and 30% of previous studies, respectively (Przeworski *et al.* 2000).

**Table 5:** Human nucleotide diversity and Tajima’s *D* at *NR2E1*

Population	*n*	*S*	θ_W_ (±SD)	π (±SD)	*η*_S_	Tajima’s *D*
Africa	36	12	0.00045	0.00024	8	−1.45
			(0.00018)	(0.00004)		
Americas	28	6	0.00024	0.00029	0	0.61
			(0.00012)	(0.00005)		
Asia	18	6	0.00027	0.00027	0	−0.36
			(0.00014)	(0.00006)		
Europe (C)	24	3	0.00013	0.00017	1	0.83
			(0.00008)	(0.00002)		
Europe (N)	34	7	0.00027	0.00027	1	−0.04
			(0.00013)	(0.00004)		
Middle-East	26	7	0.00029	0.00022	5	−0.75
			(0.00014)	(0.00003)		
Oceania	22	5	0.00022	0.00020	0	−0.18
			(0.00004)	(0.00004)		
Total human	188	21	0.00057	0.00026	11	−1.50
			(0.00017)	(0.00002)		

*n*, number of alleles; *η*_S_, number of singleton mutations; *S*, number of segregating sites.

### Evidence of non-neutral evolution at NR2E1

Genes that have been implicated in severe cortical malformations, including *ASPM* (Evans et al. 2004b) and *Microcephalin*(Evans et al. 2004a), show robust molecular signatures of positive Darwinian selection; consequently, the identification of signatures of selection in candidate neural genes such as *NR2E1* may strengthen their proposed role in human cortical disorders. To elucidate the human molecular evolution of *NR2E1*, we first used the nucleotide diversity measures θ_W_ and π to calculate Tajima’s *D* (Tajima 1989) (Table 5). Positive and negative values of this test correspond to departures from the neutral expectations of molecular evolution. We obtained a negative value for Tajima’s *D* in the ethnically diverse population, which is consistent with another report that obtained a negative Tajima’s *D* at *NR2E1* (Stephens *et al.* 2001).

To further evaluate the role of natural selection at *NR2E1*, we used the ancestral and derived states of each variant from the ethnically diverse population to perform three additional tests of molecular neutrality: Fu and Li’s D* (Fu & Li 1993), Fu and Li’s F* (Fu & Li 1993) and Fay and Wu’s *H* (Fay & Wu 2000). We obtained statistically significant negative values for Fu and Li’s D* and F* (Table 6), which may indicate genetic hitchhiking or background selection. However, based on these tests alone, we cannot exclude the possibility that demographic factors such as population bottlenecks may also explain deviations from neutrality observed at *NR2E1* (Fu 1997; Kreitman 2000).

**Table 6:** Neutrality tests using chimpanzee as outgroup

Population	Fu and Li’s D^*^	Fu and Li’s F^*^	Fay and Wu’s *H*
Africa	−2.77†	−2.79‡	0.89
Americas	1.27	1.26	1.14
Asia	1.33	1.11	0.86
Europe (C)	−0.24	0.07	0.31
Europe (N)	1.24	1.13	0.77
Middle East	−1.97	−1.81	0.69
Oceania	1.22	0.96	0.19
Total human	−3.08†	−2.63‡	0.98

† *P* < 0.02.

‡ *P* < 0.05.

### Human-specific NR2E1 sites identified

Insight into the evolution of human-specific traits, such as enlarged cerebral cortex, may be gained by the identification of human-specific sites (i.e. nucleotides that are fixed among all humans but absent from non-human species) (Enard & Paabo 2004). To identify such sites, we aligned 6137 bp of human and non-human primate coding and non-coding sequences. We identified 26 human-specific (divergent) sites (data not shown). Of these 26, five resided within functional (i.e. exons) or putatively functional (i.e. evolutionarily conserved non-coding) regions of *NR2E1*: one synonymous coding variant and four putative regulatory variants (Table 7). We extended our analysis to mouse and determined that four divergent sites still remained (the 3′-UTRs between human and mouse *NR2E1* could not be aligned) (Table 7). To determine whether these four variants may disrupt or create TFBS, we performed *in silico* TFBS analyses. We did not detect any alterations of TFBS for transcription factors expressed in the brain.

**Table 7:** *NR2E1* sites that are fixed among all humans but differ in non-human species

Region	Location* (bp)	Humans†	Great apes‡	Old world monkeys§	Mouse¶
CE11A	−2994	G	A	A	A
5′-UTR	−542	T	C	C	C
5′-UTR	−498	A	T	T	T
Exon 4††	9843	A	T	T	T
3′-UTR	21090	C	T	T	na

* Numbering adopted from Antonarakis *et al*. (1998), where A of the initiator Met codon in exon 1 is denoted nucleotide +1. Human genomic *NR2E1* sequence: NCBI AL078596.

† includes all humans examined (African, Asia, Americas, Europe, Middle East, Oceania).

‡ includes all chimpanzees, gorillas, orangutans examined.

§ includes all rhesus and Japanese macaques examined.

¶ na, orthologous region does not align with human sequence.

†† CCA_(Pro) to CCT_(Pro).

### NR2E1 haplotype and LD structure provide effective tools for disease-mapping studies

To inform future association and linkage-based studies of *NR2E1* in disorders of brain and behavior, we elucidated haplotype structure and LD using a subset of the 21 novel variants identified in our analyses of ethnically diverse and unaffected humans. To characterize the haplotype structure of human *NR2E1*, we inferred haplotypes using bi-allelic variants whose MAFs were 5% or greater. Genotypes of all markers were in Hardy–Weinberg equilibrium (data not shown). For each individual, we inferred haplotypes (Fig. 2a) and estimated the population haplotype frequencies for all seven human populations (Fig. 2b). We also typed a 12-allele CA-repeat in the 3′-UTR (dinucleotide repeat range 17–31; data not shown). We then re-constructed haplotypes using the CA-repeat data and the five most common haplotypes (Fig. 2c). Our characterization of haplotype structure in *NR2E1* identified five haplotypes and CA-repeat alleles that would be useful for disease-mapping studies.

To empirically estimate the degree of non-random association between *NR2E1* variants, we calculated LD using two statistics: Lewontin’s coefficient |*D’*| and Pearson’s correlation *r^2^* (Ardlie *et al.* 2002). We used all the variants with MAFs equal to or greater than 5% except indel variant 17 for technical reasons (see *Materials and methods*). Despite our markers being only a few kilobases apart, in most cases we observed weak LD in this region (data not shown); however, we note that small sample sizes may underestimate the extent of significant LD. The only substantial LD in *NR2E1* was between variants 2 and 7 (|*D’*|= 0.894; *r^2^*= 0.880; Fisher *P* < 0.001, significant using the conservative Bonferroni correction). We also examined the relationship between LD and distance using both |*D’*| and *r^2^*, which indicated a general decrease in the level of LD with increasing distance (data not shown).

## Discussion

The present study represents the first genetic report of *NR2E1* in clinical samples. In addition, it provides the most comprehensive evolutionary study of *NR2E1* reported to date. Our studies of *NR2E1* are noteworthy in several respects. First, we used a direct resequencing approach, which is the most reliable, complete and impartial means of mutation and polymorphism discovery; however, one limitation of using this approach alone is its inability to distinguish between homozygosity across loci vs. large deletions. Second, our experiments were designed to identify candidate mutations and polymorphisms in both coding and key non-coding regions, such as evolutionarily conserved sequences that may harbor functionally important and disease-causing variants (Drake *et al.* 2006). Third, we studied a diverse collection of human genomic DNAs representing the world’s major continental populations as a means to thoroughly assess the natural genetic variation at this locus.

Our candidate mutation screen demonstrated that protein-coding mutations in *NR2E1* do not contribute to cortical and behavioral abnormalities in the patients examined here. In addition, we detected individuals homozygous across the *NR2E1* locus, but given the overall lack of variability at the locus and the fact that these cases were not enriched in the patient sample, this data does not argue for the presence of large deletions. We did identify and characterize seven candidate non-coding mutations and four candidate functional polymorphisms. Of particular interest is patient LR00-144, who is a compound heterozygote for candidate *NR2E1* mutations, which is consistent with the recessive inheritance of the cortical phenotype in *Nr2e1^−/−^* mice. Strikingly, patient LR00-144 harbored three of the seven candidate mutations. The chances of observing three candidate mutations in a single patient are extremely rare [(7 candidate mutations/56 patients)^3^= 1.9 × 10^−3^]. The g.-1767G>T candidate mutation identified in this patient is predicted to create two transcription factor binding sites (TFBS). One of these is for the zinc finger protein insulinoma-associated 1 (*IA-1*), which is present in fetal brain tissue and functions as a transcriptional repressor during neuronal development (Breslin *et al.* 2002). *IA-1* binding sites have been identified in the 5′-flanking regions of several genes including *Pax6* and *NeuroD/β2* (Breslin *et al.* 2002). The g.-1767G>T substitution is also predicted to create a neural-restrictive-silencer element (*NRSE*). *NRSE* motifs are known to bind the neural-restrictive silencer factor (*NRSF*) that functions as a transcriptional repressor of multiple neuronal genes such as *NR2B*, which contains five *NRSE* motifs in its 5′-flanking region (Qiang *et al.* 2005). The highest expression of *NRSF* is observed in the mouse embryonic cortex at E14, but is also detected in the adult mouse brain as well as in cultured cortical neurons (Qiang *et al.* 2005). Taken together, the creation of at least two transcriptional repressor binding sites in the proximal promoter of *NR2E1* in patient LR00-144 supports the proposal that the g.-1767G>T candidate mutation could contribute to the cortical phenotype and severe mental retardation observed in this patient.

Patients LR00-204 and LR03-277 also represent compound heterozygotes for patient variants of *NR2E1*. Interestingly, LR00-204, who had microcephaly, was also diagnosed with optic nerve hypoplasia, a phenotype observed in *Nr2e1^−/−^* mice (Young *et al.* 2002; Yu *et al.* 2000). The specific pair of candidate functional polymorphisms observed in each patient was absent in all controls examined; thus, these particular combinations of alleles were specific to cortical disorders. Each of these variants may act through a hypomorphic mechanism that involves reduced levels of *NR2E1* transcription. Such a mechanism is supported by the demonstration that *Nr2e1^+/−^* mice show altered neurogenesis early during cortical development (Roy *et al.* 2004), which indicates dosage sensitivity for *Nr2e1*.

Patients 8348 and LR01-148 harbored candidate mutations g.8213T>C and g.20765C>A, respectively. Parents of both these patients were unavailable to study; consequently, we cannot exclude the possibility that both these variants may represent *de novo* mutations. The candidate mutation g.8213T>C identified in intron 3 in patient 8348 is predicted to abolish a binding site for *OCT1*, a regulator of neuronal differentiation and is also predicted to create binding sites for *BRN5*, another regulator of neuronal differentiation and *PAX6*, a regulator of neuronal proliferation and fate. The major allele T at this site is conserved between human and *Fugu*, which strengthens the proposal that a nucleotide substitution at this site may be pathogenic.

Finally, the two candidate mutations g.-1726C>A and g.14718C>T identified in patients LR02-304 and LR01-194, respectively, may also underlie cortical disorders. However, as each was also present in a parent, we propose a multigenic mechanism underlying the cortical phenotypes in each case. Such a proposal receives support from mice that are double heterozygotes for mutations at *Nr2e1* and *Pax6*, which interact genetically to alter normal forebrain development (Stenman *et al.* 2003). Both candidate mutations are predicted to alter neural TFBS and one of these (g.-1726C>A) resides in the promoter region, which together strengthens their candidacy for disease.

Our genetic diversity and evolutionary analyses of *NR2E1* in ethnically diverse humans and non-human primates will inform future *NR2E1* studies of human brain–behavior disorders. Our data indicate strong evolutionary constraint (i.e. purifying selection) in the coding region of *NR2E1* that is higher in comparison to many other genes examined for genetic diversity (Cargill *et al.* 1999; Dorus *et al.* 2004; Freudenberg-Hua *et al.* 2003) (http://genebank.nibio.go.jp/gbank/qfbase/index.html). Studying additional non-human primates would serve to further strengthen this conclusion. The implication of strong functional constraint is that any future identification of an *NR2E1* coding variant in a patient with a brain–behaviour phenotype is likely to be related to the disorder. Importantly, the striking absence of synonymous changes, which are typically considered to be selectively neutral (Enard & Paabo 2004), may suggest a functional constraint that operates at the RNA level to maintain its secondary structure or stability, as described for other genes (Capon *et al.* 2004; Chamary & Hurst 2005; Duan *et al.* 2003).

We provide evidence of adaptive evolution at *NR2E1* that may act on regulatory sites, which constitute an important class of non-coding sequences that are potential targets of Darwinian selection (Wray *et al.* 2003). We observed an excess of rare, derived *NR2E1* variants, as indicated by the significantly negative Fu and Li’s D* and F* values, which could be evidence of a ‘selective sweep’ (i.e. the rare variants have ‘hitch-hiked’ along with a variant on which positive selection has occurred) (Fay & Wu 2000). In this regard, it is conceivable that one or more of the human-specific *NR2E1* sites identified in the present study may have been fixed by positive selection in a manner similar to that proposed for *ASPM*, which is mutated in some patients with microcephaly (Bond *et al.* 2003; Kumar *et al*. 2004; Woods *et al.* 2005).

The knowledge of the genetic architecture of *NR2E1* generated in this study in ethnically diverse humans and non-human primates provides additional tools for future disease-mapping studies of brain–behavior disorders. Our results expand those of one other study that examined normal genetic architecture at *NR2E1* (Stephens *et al.* 2001); however, our analyses employed over twice as much sequence data (including evolutionarily conserved regions not previously examined) from a more diverse set of humans and non-human primate species. The identification and characterization of common SNPs, microsatellites, and haplotypes in multiple ethnic groups will benefit future association analyses of brain–behavior disorders by helping to reduce or eliminate false-positive and negative associations that can arise as a result of population stratification, which is a well established confound in human disease-mapping efforts (Freedman *et al.* 2004; Kang *et al.* 1999). We also provide the first example of a human polymorphic site that is also polymorphic for the same alleles in chimpanzee, gorilla and orangutan. Therefore, we strongly recommend that multiple non-human primate species be used to robustly infer ancestral states of human polymorphisms.

In conclusion, our analysis of human *NR2E1* has identified candidate regulatory mutations and rare putative functional polymorphisms. Future work will involve testing these alleles for abnormal function using whole-animal assessment as proposed by Abrahams *et al.* (2005). In this study, we selected patients enriched for features resembling *Nr2e1^−/−^* mice in addition to microcephaly (e.g. four patients with agenesis of the corpus callosum, one patient with optic atrophy). Future research may benefit by focusing on other region-specific malformations present in the *Nr2e1^−/−^* mice, such as preferential reduction of superficial cortical layers II and III, in an effort to enhance detection of *NR2E1* mutations. Our genetic diversity and evolutionary analyses provide the foundation to facilitate future examination of the role of *NR2E1* in additional human disorders of brain and behavior.
